# Prevalence of Low Birth Weight and Its Developmental Vulnerability Among Infants in Nepal: A Critical Review of the Literature and Future Recommendations

**DOI:** 10.31729/jnma.9112

**Published:** 2025-06-30

**Authors:** Sangita Pudasainee-Kapri

**Affiliations:** 1 School of Nursing-Camden, Rutgers The State University of New Jersey, New Jersey, USA

**Keywords:** *developmental outcomes*, *economic burden*, *impacts*, *low birthweight*, *preterm birth*

## Abstract

Limited research has focused on developmental vulnerability of low birth weight (LBW) infants in Nepal. This review analyzes the prevalence and magnitude of LBW in Nepal followed by developmental vulnerabilities and the economic impact of LBW.

This is a comprehensive review of current evidence-based literature relevant to the topic. A thorough literature search was conducted across different databases and relevant websites including CINAHL, PsycINFO, Medline, PubMed, Ministry of Health and Population, WHO, UNICEF, etc. Resources cited and kept were articles written in English and dated within the last fifteen years, except for historical and context-specific relevant materials.

Majority of research and programs are focused on improving the survival of LBW and preterm infants. There is limited research on developmental outcomes of LBW contributing to a lack of early monitoring, follow-up, and proper interventions.

Additional research and interventions targeting parents of LBW infants are needed to reduce the negative developmental consequences of LBW. This review suggests recommendations for future research and the need for education and training among healthcare professionals to improve outcomes.

## INTRODUCTION

Low birth weight (LBW) is considered an important public health problem worldwide affecting more than 20 million (15-20% of all births) infants globally ^1^. Recent statistics indicate that lower-middle-income countries (LMICs) have a significant burden of LBW with variations across regions in which more than 95% of LBW infants are born in LMICs^1^ with second-highest prevalence of LBW in Nepal compared to other South Asian countries.^2^ Evidence from developed countries indicates that LBW is associated with a range of adverse health and developmental consequences throughout childhood.^3,4,5^ Prior research indicates that LBW is a major predictor of increased infant and child mortality and morbidity and poor developmental outcomes in developing countries including Nepal.^6,7^

Nepal is one of the landlocked countries in South Asia. There is a high degree of geographic (Hill, Terai, and Himalayan) and ethnic diversity (Brahmin, Chettri, Dalit, Newar, Muslim etc.) in Nepal where mothers from Dalit and disadvantageous ethnic groups are three times more likely have LBW infants (AOR = 2.9, 95% CI = 1.2-7.1) compared to non-Dalit ethnic groups.^6^ Some hospital-based studies indicate that LBW prevalence is much higher (i.e., 15.3% to 23.6%) in Nepal than other low-middle income countries in South Asia.^6,8^ However, accurate national estimates of LBW prevalence cannot be determined in Nepal due to geographic diversity issues and logistic difficulties in recording birth weight in at-home deliveries.^9^

LBW is a topic of great importance in Nepal as it is a significant predictor of infant and child mortality, stunted growth.^10^ Additional research also indicates that LBW is associated with poor cognitive and socioemotional outcomes.^3,11-14^ Along with prematurity and intrauterine growth restrictions, several maternal and socio-economic risk factors may contribute to poor birth outcomes in Nepal.^15^ Studies from developed, high-income countries also suggest that parenting factors such as higher levels of positive maternal warmth and interactions during early childhood may stimulate the immature brain of LBW infants, thereby facilitating positive cognitive and academic achievement, increased social skills, and decreased externalizing behaviors across childhood.^3,12,16,17^ Therefore, there is a great need for training health professionals in the management of LBW babies, besides education for women of reproductive age to enhance awareness about risk factors and developmental consequences.

The purpose of this review is to analyze the situation of LBW in Nepal and provide a description of the prevalence and magnitude of LBW in Nepal and among different ethnic groups. This review critically analyzes the developmental vulnerabilities of LBW in the context of other nations and Nepal with evidence from current literature followed by economic impact of LBW. Finally, the importance of education and training for healthcare providers and parents of LBW infants followed by recommendations for policy and practice will be discussed. This review has significance for other developing countries and LMICs since the policy, education, and training recommendations are useful for other LMICs (including, India, Pakistan, Bangladesh, etc.) where there are similar socioeconomic structures with a high burden of LBW.

## METHODS AND RESEARCH SELECTION

A literature review was carried out to identify current evidence and gaps on LBW, preterm birth, and associated developmental outcomes in Nepal and other countries. The search strategy included CINAHL, PubMed, PsycINFO, Cochrane, and Google Scholar; gray literature was sourced from databases of WHO, UNICEF, and government of Nepal-related websites. Only English language empirical articles related to outcomes of LBW were included for review from a period of last 10-15 years for both developed and low-middle-income countries. The key words used for searching included LBW, preterm birth, prevalence, developmental outcomes, economic burden, evidence- based interventions, and healthcare costs. Relevant historical data specific to Nepal prior to 15 years were also included. A total of 580 records were initially identified across databases. After title and abstract screening, 132 articles were considered potentially relevant. Following full-text review and exclusion of duplicates and irrelevant content, 56 articles were included in the final synthesis.

Peer-reviewed journals, Ministry of Health documents, Nepal Demographic Health Survey reports, and publications from WHO/UNICEF, were reviewed as guided by Grant and Booth (2009).^18^ Findings compare evidence between developed and low-middle-income countries and thus inform policy recommendations for Nepal in reducing morbidity and mortality among LBW children. Qualitative studies, editorials, and commentaries were excluded in this review to maintain a focus on empirical evidence. Although this is not a systematic review, a basic quality appraisal of included studies was conducted using adapted criteria from the Mixed Methods Appraisal Tool and Critical Appraisal Skills Programme checklists.

## LOW BIRTH WEIGHT: CONSEQUENCES, MAGNITUDE, AND PREVALENCE

LBW is defined as a newborn's birth weight of less than 2500 grams (5.5 lbs) irrespective of their gestational age.^5^ The major causes of LBW include preterm birth (infant born with <37 completed weeks of gestation), intrauterine growth restriction (IUGR: a lack of proper growth during intrauterine life due to birth defects, maternal health conditions, or problems with the placenta), or both preterm and IUGR.^19^ LBW infants due to IUGR are also known as small for gestational age (SGA: defined as birth weight for given gestational age less than the 10^th^ percentile). LBW infants represent a heterogeneous group of term and preterm infants who carry significant risks in both health and developmental outcomes across the lifespan. LBW infants who are also born premature are at an even higher risk for poor neonatal and adverse developmental outcomes (Table 1).^20^

**Table 1:** Summary of Key Literature on Low Birth Weight and Developmental Vulnerability

Authors name	Topic Area	Key Finding	Context
WHO, 2012; WHA 2012^5^	LBW Definition & Global Target	LBW defined as <2500g; WHA set global target of 30% reduction in LBW by 2025	Global
Ministry of Health Nepal, NDHS 2015 & 2022^9,23^	Prevalence of LBW in Nepal	LBW prevalence in Nepal 21.8% (2015); increase in institutional deliveries to 79% (2022)	Nepal
Thapa et al., 2022 (Tertiary Hospital Study)^6^	LBW Prevalence	15.3% LBW among 308 postpartum mothers, and Higher LBW prevalence among Dalits; AOR = 2.9	Nepal (Hospital- based)
Christian et al., 2014^22^	Rural LBW Burden and Nutritional Interventions	39% LBW prevalence in rural Nepal, higher IUGR incidence Micronutrient supplementation and maternal anemia management reduce LBW	Nepal (Rural)
UNICEF 2004; 2007; 2012; 2019; 2025^2,7,24,26,27^	Global LBW Prevalence, Developmental Outcomes	Nepal ranks 2nd in South Asia and 3rd globally in LBW. LBW infants have lower IQs, more behavioral and learning problems. Focus on maternal nutrition, prenatal care, safe delivery, and newborn care	Global/Nepal
de Bie HMA., 2010^31^	Cognitive Impact	Rural LBW children in Nepal show poor cognitive outcomes and reduced cortical thickness	Nepal
Ranjitkar S et al., 2019^32^	Cognitive Assessment	LBW children in Nepal scored lower on Bayley-III developmental scale	Nepal
Rikknen 2009; Vaske 2013^14,34^	Socioemotional Challenges	LBW linked to aggression, behavioral maladjustment, and later psychopathology	Global
Nordhov et al., 2012^16^	Parenting Interventions	Maternal warmth mitigates developmental delay in LBW children	Global
Pudasainee-Kapri S 2022^12^	Low Birth Weight and Socioemotional Competence	LBW is associated with poor cognitive and socioemotional outcomes.	Global
Pudasainee-Kapri S et al., 2019^3^	Low Birth Weight and cognitive competence.	Low birth weight and maternal warmth influence children's cognitive competence.	Global
Shrestha et al., 2020^10^	Predictors of Low Birth Weight	Education, occupation, gestation, delivery mode, and weight affect LBW.	Nepal
Upadhyaya et al., 2019^13^	Cognitive and motor outcomes in LBW	LBW (< 2000 g birth weight) have substantial cognitive and motor impairment compared to Normal birth weight.	Asia

Despite implementation of the evidence-based interventions, Nepal has the second-highest prevalence of LBW in South Asia and the third-highest prevalence in the world.^7^ Although infants born with LBW have declined from 27.2% in 2000 to 22.6% in 2012 in Nepal, the incidence of LBW has remained fairly constant over the last decade (21.8% in 2015).^7,21^ A recent tertiary hospital-based study among 308 postpartum mothers in Nepal found the prevalence of LBW to be 15.3% or one out of seven infants.^6^ Along with geographic variations in a LBW, ethnic diversity existed in Nepal with Dalit and disadvantageous ethnic groups having higher odds of having LBW infants (AOR = 2.9, 95% CI = 1.2-7.1) compared to non-Dalit ethnic groups.^6^ The prevalence of LBW was much higher in one of the rural community-based studies in Nepal, and 39% of infants were born LBW with a higher percentage of IUGR infants.^22^ Although the health situation in Nepal has substantially improved over the years, the higher prevalence of LBW may contribute to higher mortality rates in neonates, infants, and children under five years of age and persistent long-term adverse health and developmental consequences among children in Nepal.

A major challenge in obtaining accurate national estimates of LBW in Nepal is due to logistic difficulties in recording birth weights, as almost 19 % of deliveries are still conducted at home without any medical assistance.^23^ A large volume of data on birth weight is available from hospitals and health centers, but nearly three-quarters of newborns in Nepal are not weighed at birth. Thus, a lack of comparable data makes examining progress on LBW difficult.^19,24^ The estimates of LBW prevalence only from the sample of measured birth weight and or imputation of missing data which may however introduce bias and or underestimate its actual prevalence.^25^ Hence, the most available data on birth weight is not representative of the total population. Instead, this data mostly represents those from higher socioeconomic groups whose infants are born in hospital or health centers and those living in urban areas. Overall, data obtained from urban or hospital-based studies and or small-scale tertiary level hospital-based studies may not be comparable to rural settings. The rates of institutional deliveries (79% per 2022 NDHS survey) and deliveries conducted by skilled healthcare providers (80%) have increased in Nepal which may strengthen national estimates of LBW measurement.^23^ Thus, Additional research is needed to accurately estimate the national prevalence of LBW in Nepal as well as disparities across geographic and socioeconomic groups.

## DEVELOPMENTAL OUTCOMES OF LOW-BIRTH-WEIGHT INFANTS

LBW generally increases the risk of neonatal and infant mortality and childhood morbidity compared to normal birth weight (NBW: birthweight >2500 grams) infants.^5,26^ LBW infants have a 40-fold increased risk of neonatal mortality, increased incidence of childhood morbidity, 50% greater chance of having serious development problems (e.g., learning disabilities and mental retardation), and 5-10 point lower Intelligent Quotients (IQs).^27^ The consequences of LBW may extend into adulthood as LBW children may suffer from a variety of chronic illnesses including diabetes, hypertension, and obesity.^21,27^ The survival rate of LBW infants has improved in recent years with advancements in maternal-fetal medicine and neonatal intensive care in Nepal resulting in a significant reduction in neonatal and infant mortality as well as childhood morbidity. There is a greater risk for ongoing vulnerabilities in multiple domains of developmental outcomes particularly developmental delays, poor cognitive outcomes, and socio-emotional competencies across childhood and may extend into adulthood.

The first 1,000 days, from conception up to the end of the second year of life, are particularly critical for children's cognitive development.^28^ Biological adversity and/or psychosocial factors during this critical period may lead to impairment in the structural and functional development of the brain.^29^ Similarly, available studies from high-income countries suggest that LBW and pre-term infants are at higher risk for poor brain development during pregnancy and/or failure of catch-up growth during the infancy period and a reduced cortical thickness.^31,32^ These biological vulnerabilities have an adverse impact on multiple domains of cognitive outcomes including intelligence, executive and motor function during 7-9 years of age among rural community children born LBW and SGA in Nepal.^22^ A systematic review among LBW children in South Asia indicates that infants weighing <2,000 g at birth have substantially more cognitive and motor impairment compared to NBW infants. ^13^ Similarly, LBW infants score significantly lower on cognitive composite scores measured by the Bayley III Developmental Assessment compared to NBW infants in Nepal Developmental issues, increased risk for poor intellectual performance (reading, spelling, and mathematics), and have also been found among moderately low birth weight (MLBW: birth weight 1,500-2,499 grams) infants are at increased risk for socioemotional difficulties at age nine in moderately low birth weight infants in the US.^3,12,32^

Additionally, the development of socio-emotional competence is another critical area for these at-risk children when they enter school due to the complex interactions with teachers and peers. Although the magnitude of the impairment varies across studies and subgroups of LBW and preterm children, there is agreement that they are generally less likely to be socially competent and more likely to experience emotional and behavioral problems than their NBW counterparts.^12^ Literature on behavioral competence also suggests that LBW children are at increased risk of developing externalizing problem behaviors such as aggression, delinquency, and hyperactivity throughout childhood and behavioral maladjustment and psychopathology in later life.^11,14,33,34^ Although the mechanism of these relationships is not well understood in the literature, they are consistent with the developmental vulnerability hypothesis.^35^ The diverse biological and or health-related risks, adverse home and social environment, and the quality of early parenting factors may have a persistent impact on the developing brain of the infant leading to increased cognitive, academic, and behavioral difficulties among LBW at risk children.^3,11,12^ Additionally, some research from high-income countries indicates that LBW and preterm children are also more susceptible to difficult temperament (more likely to exhibit higher sensory thresholds, less adaptable, and more intense) and more challenging to manage than NBW and or full-term counterparts during early childhood years, resulting in slower adjustments to environmental changes, and increased likelihood of behavioral problems.^36,37^

Similarly, the developmental vulnerability hypothesis indicates that LBW children are more sensitive to adverse environmental situations due to their temperamental characteristics, thus requiring higher levels of maternal warmth and sensitivity during their critical years of development to achieve positive outcomes.^3,4,17,35,36^ Several studies indicate that early positive parenting such as maternal warmth may moderate the adverse impacts of LBW on cognitive and behavioral competence.^3,4,38^ Findings of US-based intervention studies demonstrate that enhancing maternal warmth, stimulation, and positive interactions as well as stimulating home environments can have a positive impact in multiple areas of development among LBW infants.^16,39^ This includes better cognitive performance, IQ scores, and math achievement during preschool years as compared to the control group of LBW children.^40^

The development of cognitive and socioemotional competence in childhood has a significant impact on later health and well-being. The majority of published studies on neurocognitive and behavioral outcomes of LBW are from high-income countries, while there is a lack of evidence in Nepal. Studies in LMICs suggest that infants born LBW are at high risk for poor developmental outcomes compared to NBW children.^13,41^

## ECONOMIC IMPACT

Although no exact data are available for Nepal, it is clear that the high rates of mortality, morbidity, and developmental delay associated with LBW and/or preterm LBW impose an enormous burden on national health, education, and social services, as well as among families and society in general. To date, there is a lack of empirical research that has estimated the economic implications of LBW in the context of Nepal. Longer hospital stays and extreme LBW status contributes to substantially higher medical costs in which the costs of hospitalization per day in developed countries range from $500 to 1250 to 2500 as reported across multiple studies.^42^ Although total medical costs of care vary across LBW categories (i.e., SGA, preterm LBW, or very/extremely LBW - birthweight <1500 grams), LBW infants in general have higher medical costs than NBW and full-term infants due to severe medical conditions and longer hospitalizations.^43^

A recent study in India (a LMIC) indicated that the median costs for direct medical care and NICU admission was USD 331 (i.e., INR 21,430).^44^ The cost of hospitalizations and ongoing medical treatment are significantly higher in developed countries compared to LMICs countries. Health insurance policies generally offset the burden of NICU cost and support for infants' medical cost in developed countries, whereas it usually comes from out-of-pocket expenditure for parents in LMICs countries.^42^ This can lead to a substantial cost burden for families in Nepal resulting in putting those infants in at a great disadvantage for receiving high-quality care during the neonatal period and throughout infancy and childhood.^45,46^

Although the survival of LBW and LBW preterm infants or extreme LBW infants has been improved with the advancements in technologies and treatments,such as the introduction of CPAP, mechanical ventilation, and exogenous surfactant administration since the early 2000s, these children may be at increased risk for longterm consequences in health and multiple domains of development including cognitive, learning, and socio-emotional competence.^42^ Thus, they require longterm evaluation, follow-up, and monitoring to detect and address health and developmental problems promptly. The economic costs of preterm LBW infants are higher when the gestational age at birth is lower since they tend to need longer initial NICU stays and additional medical costs incurred throughout the childhood.^47^ Hence, LBW can result in substantial costs even after initial hospital discharge. Investment in education and training can substantially reduce the overall costs incurred from LBW infants over time. Moreover, healthy children are assets for a nation's overall economic and social development.

## DISCUSSION

LBW is a major public health problem in Nepal, associated with higher morbidity and mortality and poor developmental outcomes. Since more than two-thirds of infants are born at home or at small private clinics and are not reported in official figures, logistic difficulties in recording birth weight contribute to the underestimation of the prevalence of LBW in Nepal. ^23^ Also, the lower-income and higher-risk groups are least likely to be included in hospital or urban-based data sets. The determinants of LBW vary across diverse geographic, socioeconomic, and ethnic groups, thus a detailed analysis of potential contributing factors is required in Nepal. Overall, improvement of dietary intakes among women, adoption of measures to reduce the risks of preterm birth, and supplementation of iron and folic acid during pregnancy are considered some evidenced-based strategies to reduce the prevalence of LBW in Nepal.^5,48^ LBW children, in general, need frequent monitoring, developmental follow-up, and early intervention to minimize the risk of poor developmental outcomes. To address LBW issues, further research and educational interventions, pathways for predicting LBW, and guidelines for measuring developmental outcomes are warranted in Nepal Despite consistent scientific and technological interventions that have contributed to improved survival and decreased mortality of LBW infants over the last two decades, LBW due to IUGR and preterm birth continues to be one of the leading causes of persistent long-term adverse consequences in multiple areas of development including developmental delay, cognitive impairment, and socio-emotional difficulties. However, there is limited research regarding the longterm consequences and impact of LBW in Nepal. Also, there has been insufficient attention in providing training and education for healthcare professionals regarding the developmental vulnerability of LBW infants.

## EDUCATION, TRAINING, AND INTERVENTIONS

Various organizations have set goals for reducing the prevalence of LBW and child mortality in Nepal. The Safe Motherhood Program (SMP) is one of the priority programs in Nepal that was designed to reduce maternal and neonatal mortality. The SMP contributes to LBW reduction and infant survival by focusing on maternal nutrition, prenatal visits, basic and comprehensive essential obstetric care services, healthcare-assisted deliveries, and safe newborn care.^30^ Despite targeted programs and policies during pregnancy and the early postpartum period, there is a lack of continuous growth monitoring and developmental follow-up of LBW infants from skilled healthcare professionals in Nepal.

In 2012, the World Health Assembly (WHA) endorsed a comprehensive implementation plan on maternal and child nutrition along with six global nutrition targets including a 30% reduction of LBW by the year 2025.^5^ The current rate of progress toward the reduction of LBW rates in Nepal would have to fall well below the annual reduction rates of 2.7% to meet this target. Similarly, the reduction of LBW and its consequences would considerably contribute to the Millennium Development Goals (MDGs:1990 - 2015) for reducing child mortality. ^7^ Monitoring the progress towards LBW is currently reiterated under the third Sustainable Development Goals (SDGs: 2016 - 2030) "Health and Well-being". To end preventable deaths among children, the SDGs goal related to neonatal and under-five mortality rates in Nepal is 12 to 20 deaths per 1,000 live births by 2030.^49^ Thus, reduction of LBW and preterm birth are key indicators of progress to achieve the overall health and well-being of infants and children. There have been considerable reductions in maternal, neonatal, and under-five mortality rates in Nepal in recent decades, however, the progress toward meeting targets has been slow and insufficient.

Women of reproductive age, pregnant women, and their families should be included in educational interventions regarding factors contributing to LBW and its potential short-and long-term effects on children. Additionally, maternal weight gain and nutritional intake are closely linked to fetal development and subsequent birth weight, education about health, diet and adequate weight gain during pregnancy are essential for the overall reduction of LBW prevalence and to achieve global nutrition targets.^5,21,50^ Early and routine prenatal visits allow healthcare providers to identify potential risk factors in pregnant women and provide early intervention to promote optimum outcomes in Nepali women.

There is also a lack of skilled and competent healthcare providers for the provision of quality perinatal services and identification of high-risk conditions for mother and fetus in the rural areas requiring timely management and referral, instead most available resources are located in urban areas and tertiary level hospitals in Nepal. Despite the consistent efforts of governmental and non-governmental organizations (such as UNICEF, WHO, and USAID) to reduce the proportion of LBW and improve infant outcomes, the progress has been slow. Thus, evidence-based interventions are needed to reduce the prevalence and associated mortality, morbidity, and long-term consequences of LBW.^5^ Such interventions may include peri-conceptual folic acid supplementation, supplementation of micronutrients for women of reproductive ages, the provision of adequate nutrition for adolescent girls, promotion of smoking cessation and education on harmful substance use during pregnancy and after childbirth, and early intervention and consistent monitoring for at-risk infants.^19^ Since this review may not directly inferred the effectiveness of such interventions and programs, future research should explore the effectiveness and challenges or inhibiting factors of existing interventions to improve maternal and birth outcomes in Nepal.

Improvement in education and training at various levels is thus an important prerequisite to achieve the reduction targets of LBW and improvement in health and developmental outcomes of the vulnerable child. Some effective strategies like: Several studies have outlined evidence-based interventions to reduce LBW and preterm births in low- and middle-income countries, including micronutrient supplementation, maternal education, and strengthened healthcare systems.^51^ Similarly, the long-term effects of SGA and preterm birth on undernutrition in childhood were presented, and early interventions were called for targeting vulnerable infants.^52^ Another study from Nepal emphasized that maternal anemia, enhancement of prenatal care, and health education for pregnant women will help in reducing the prevalence of LBW.^53^

Community members can be trained to measure and record birth weights during home deliveries for early identification and management of infants with LBW.^54^ Improvement of home visitation services coupled with decreases in geographic barriers and socioeconomic could extend quality health services to women and vulnerable infants.^55^ Directed at women of childbearing age and parents of LBW babies, emphasize the best possible long-term developmental outcome. Optimization of health status in women with chronic medical conditions; healthier behaviors during pregnancy; reduction of socio-demographic and environmental risk factors.^56^

## CONCLUSIONS AND POLICY RECOMMENDATIONS

Low birth weight is a major public health problem in low-middle income countries including Nepal that is associated with higher morbidity and mortality and poor developmental outcomes across multiple domains. LBW infants may not only suffer from a range of short- and longterm adverse health and developmental consequences but also contribute to increased healthcare costs and disease burden. Although Nepal’s government in coordination with different organizations have developed and implemented programs and policies to improve the maternal and infant wellbeing consistent with SDGs goal, LBW is still a major challenge in Nepal. Thus, this review has addressed the need for additional research and interventions to reduce the risk factors of adverse birth outcomes and to decrease the long and record birth weights during home deliveries for early identification and management of infants with LBW.^54^ Improvement of home visitation services coupled with decreases in geographic barriers and socioeconomic could extend quality health services to women and vulnerable infants.^55^ Directed at women of childbearing age and parents of LBW babies, emphasize the best possible long-term developmental outcome. Optimization of health status in women with chronic medical conditions; healthier behaviors during pregnancy; reduction of socio-demographic and environmental risk factors.^56^

## CONCLUSIONS AND POLICY RECOMMENDATIONS

Low birth weight is a major public health problem in low-middle income countries including Nepal that is associated with higher morbidity and mortality and poor developmental outcomes across multiple domains. LBW infants may not only suffer from a range of short- and long-term adverse health and developmental consequences but also contribute to increased healthcare costs and disease burden. Although Nepal's government in coordination with different organizations have developed and implemented programs and policies to improve the maternal and infant wellbeing consistent with SDGs goal, LBW is still a major challenge in Nepal. Thus, this review has addressed the need for additional research and interventions to reduce the risk factors of adverse birth outcomes and to decrease the long-term developmental consequences of children born with LBW. In addition, the need for training and educational initiatives to healthcare professionals for better-quality services and improved outcomes for the perinatal population and at-risk infants in Nepal are imperative. Although, the extensive focus of education and other interventions emphasize measures toward improving the survival of LBW and pre-term infants, there is a lack of attention on the developmental consequences and long-term sequelae of being born LBW. This review suggests some important implications for policy and practice to improve the quality of care and design education and training for healthcare providers and patients/families to enhance positive birth outcomes and minimize the short and long-term developmental consequences of LBW.

Policymakers and public health professionals can support the design and implementation of early intervention programs and policies that will enhance positive parenting and optimal developmental outcomes for at-risk children. Based on the findings of extant studies in LMICs and developed countries and intervention program for LBW preterm infants such as Infant Health and Development Program, investing in programs for LBW and pre-term born infants and their families may have substantial implications for long-term health and optimal developmental outcomes among children in Nepal. These findings collectively highlight the need for investment in research and training to accelerate progress towards an understanding of key factors contributing to LBW and preterm birth and corrective actions for the reduction its long-term developmental consequences of those infants. Additional research into the feasibility and effectiveness of such interventions is required in Nepali context. These recommendations are useful for other developing countries and or South Asian countries such as India, Pakistan, Bangladesh etc. where we can find comparable sociodemographic and economic structures and high burden of LBW and preterm birth.

It is vital to invest in education and training programs to reduce LBW; not only for ethical reasons to avoid suffering and hardship for the children and their families but also to reduce the cost of healthcare and overall economic burden. Further innovative research is needed to assess the feasibility and scientific validity of these recommendations.
